# Cardiac tissue engineering: an emerging approach to the treatment of heart failure

**DOI:** 10.3389/fbioe.2024.1441933

**Published:** 2024-08-15

**Authors:** Hossein Rayat Pisheh, Fatemeh Sadat Nojabaei, Ahmad Darvishi, Ali Rayat Pisheh, Mahsa Sani

**Affiliations:** ^1^ Department of Tissue Engineering and Applied Cell Sciences, School of Advanced Medical Sciences and Technologies, Shiraz University of Medical Sciences, Shiraz, Iran; ^2^ Student Research Committee, Shiraz University of Medical Sciences, Shiraz, Iran; ^3^ Department of Medical Biotechnology, Faculty of Allied Medicine, Iran University of Medical Science, Tehran, Iran; ^4^ School of Pharmacy, Shiraz University of Medical Sciences, Shiraz, Iran; ^5^ Department of Biology, Payam Noor University (PUN), Shiraz, Iran; ^6^ Shiraz Institute for Stem Cell & Regenerative Medicine, Shiraz University of Medical Sciences, Shiraz, Iran

**Keywords:** cardiomyocytes, regenerative medicine, heart failure, tissue engineering, decellularized matrix, scaffold contractility, electrical conduction, metabolic balance

## Abstract

Heart failure is a major health problem in which the heart is unable to pump enough blood to meet the body’s needs. It is a progressive disease that becomes more severe over time and can be caused by a variety of factors, including heart attack, cardiomyopathy and heart valve disease. There are various methods to cure this disease, which has many complications and risks. The advancement of knowledge and technology has proposed new methods for many diseases. One of the promising new treatments for heart failure is tissue engineering. Tissue engineering is a field of research that aims to create living tissues and organs to replace damaged or diseased tissue. The goal of tissue engineering in heart failure is to improve cardiac function and reduce the need for heart transplantation. This can be done using the three important principles of cells, biomaterials and signals to improve function or replace heart tissue. The techniques for using cells and biomaterials such as electrospinning, hydrogel synthesis, decellularization, etc. are diverse. Treating heart failure through tissue engineering is still under development and research, but it is hoped that there will be no transplants or invasive surgeries in the near future. In this study, based on the most important research in recent years, we will examine the power of tissue engineering in the treatment of heart failure.

## Graphical Abstract

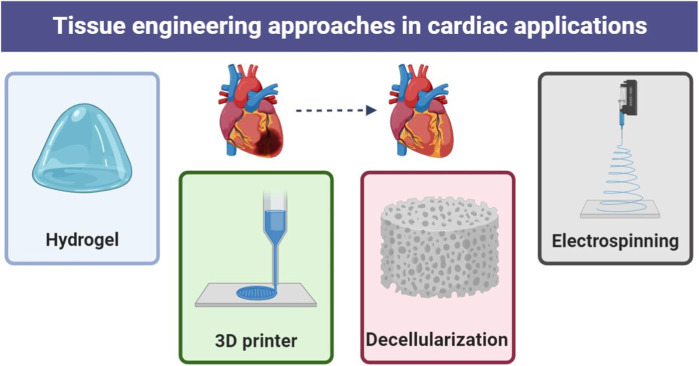

## 1 Introduction

According to the American Heart Association (Uretsky and Wolff, 2021; Yan et al., 2022), heart failure, which is characterized by the inability of the heart to pump blood effectively, affects approximately 6.5 million people in the United States. The prevalence of heart failure increases with age and approximately 10% of people over 65 years of age suffer from the disease (Pujante Alarcon et al., 2022). Various factors such as heart attack, cardiomyopathy and heart valve disease can be the cause of this disease (Molnar et al., 2023). In general, treatment for heart failure involves lifestyle changes, medications (including diuretics, ACE inhibitors, ARBs, beta-blockers, and aldosterone antagonists), devices (pacemakers, implantable cardiac defibrillators (ICDs), and left ventricular assist devices (LVADs)), or surgery are invasive (Ravichandran et al., 2021; Obi et al., 2023; Tanawuttiwat et al., 2023).

The methods mentioned are associated with many complications and risks, but on the other hand they also entail many costs and restrictions. While current treatment options aim to relieve symptoms and slow disease progression, the search for a lasting cure continues. Tissue engineering is a new science that, with its potential to regenerate or repair damaged cardiac tissue, appears to be a promising way to address this global challenge (Hendrickson et al., 2021; Ciolacu et al., 2022). The principles of tissue engineering are based on the three fundamentals of scaffolds, cells and growth factors. There are multiple ways to use each. Tissue engineering reduces the need for organ transplants and the side effects of medications. This science is currently at an early stage of development, but recent developments show that tissue engineering can significantly contribute to the treatment of various diseases in the future (Lin et al., 2023; Tolabi et al., 2023).

This article, based on studies from recent years, looks at the power of tissue engineering in heart failure and examines its various approaches and treatment options. We discuss the use of stem cells and biomaterials to create functional 3D heart constructs. We also look at preclinical successes observed in the laboratory and analyze the potential to improve cardiac function.

## 2 Cardiac structure

The human heart serves as the epicenter of existence, providing nutrition and oxygen to maintain overall blood flow throughout the various organs of the body. It actively expels waste products while working like a pump and houses four chambers (called the left and right atria and left and right ventricles) and four valves (specifically mitral valve, aortic valve, tricuspid valve). and pulmonary valve) (Johnson et al., 2021; Pugliese et al., 2022). Contraction of the left ventricle (LV) or right ventricle (RV) moves blood into either the systemic or pulmonary circulation. The proper functioning of the human heart is regulated in a complex manner by a mechanical feedback mechanism (MEF). These remarkable structures, known as cardiomyocytes, are electrically stimulated to maintain the normal physiological processes of the heart (Edward et al., 2023). Cardiomyocyte elongation, which affects the heart’s electrical signals, has been described as one of the main links between cell elongation and its biological effects. Stretch-activated channels are also involved in the mechanical feedback mechanism (MEF). The heart’s natural pulse beat changes the arrangement of the heart muscle cells and the sequence of cells within the heart muscle. This presents a challenge in observing changes in cells caused by both normal body functions and abnormal health conditions (Gerach and Loewe, 2024).

**FIGURE 1:**
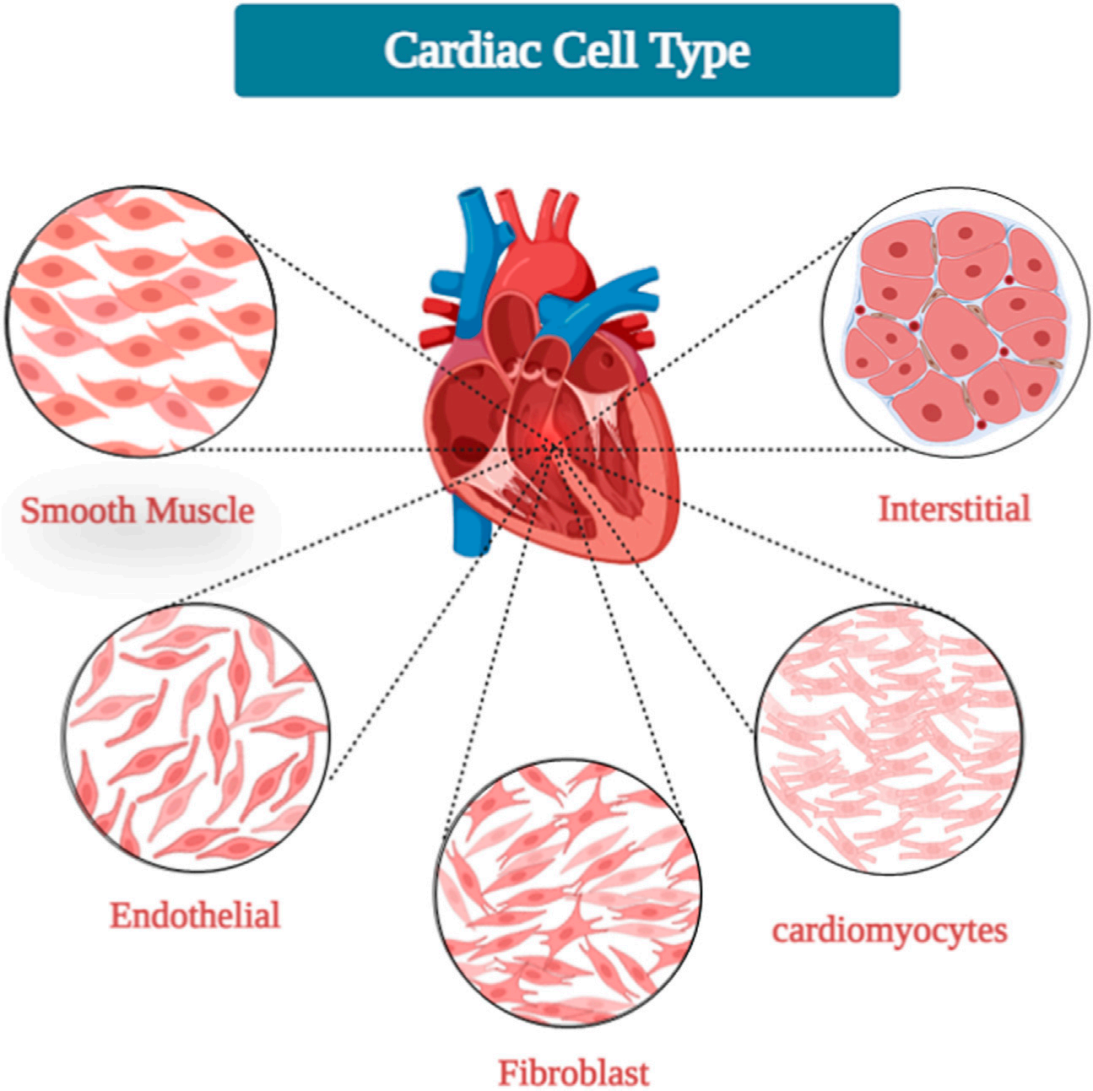
Cardiac cell types.

In the human body, all organs and tissues are a combination of different cell types. Its architecture varies depending on location, with the muscular layer of the ventricular wall, called myocardium, containing a vibrant cell community (Faber et al., 2022). There are several key cells here (Figure 1):

Cardiomyocytes: The cellular components make up about 70 percent of the volume of the ventricular wall and play a crucial role in controlling the mechanisms that ensure continuous blood circulation throughout the body. These cells monitor a sophisticated interplay of muscle contractions, electrical signals and metabolic regulation. In contrast to skeletal muscles, the branches of cardiomyocytes connect with each other and form syncytia. This combination facilitates the rapid transmission and integration of electronic signals (Bernardi et al., 2020; Nicin et al., 2022). Contraction of the sarcomere is achieved through a cycle of interactions between bridges driven by ATP, while strong contractions are generated by the conversion of electrical signals. The sarcolemma, a plasma membrane found exclusively in cardiomyocytes, is surrounded by ion channels. These channels are tasked with regulating the entry and exit of ions such as calcium and potassium while controlling electrical signals in the heart (Yamaguchi et al., 2022). Cardiomyocytes have fascinating properties and can perform on their own. This process involves an electrical depolarization wave that initiates the release of calcium from the sarcoplasmic reticulum, ultimately triggering a contraction. Gap junctions play a crucial role in establishing direct electrical connections between cardiomyocytes, enabling synchronized cardiac contractions and blood flow. The autonomic nervous system is responsible for controlling and transforming electrical signals and changes in heart rate through the use of neurotransmitters such as adrenaline and acetylcholine. Cardiomyocyte hypertrophy, fibrosis, mitochondrial dysfunction, and ion channel irregularities can have deleterious effects on contractility, electrical conduction, and metabolic balance. These factors can ultimately lead to heart failure, cardiac arrhythmias, and a variety of other cardiovascular diseases (Abi Gerges et al., 2022; Cinato et al., 2023).

Fibroblasts: Cardiac fibroblast contraction is often influenced by cardiac muscle cells, but even if they are not directly involved in contraction, they play a crucial role in maintaining muscle wellbeing. Cardiac fibroblasts make up approximately 70% of noncardiac cells in the heart and are the most abundant cell type in myocardium. They create and repair various components of the extracellular matrix (ECM), which includes collagen, elastin and proteoglycans. These factors determine the stiffness, flexibility and signaling in the heart muscle and also influence its ability to contract and conduct electrical impulses (Liu et al., 2021; Chakrabarti et al., 2023). Fibroblasts continually adapt their composition and structure to growth signals, physiological demands, and injury. This interaction determines the function of the heart muscle throughout a person’s life (Coscarella et al., 2023). Cardiac fibroblasts function as signaling cells that release growth factors, cytokines, and chemokines that affect endothelial cells and the immune system. These signals regulate numerous processes such as blood vessel formation, inflammation and excessive growth. Cardiac fiber cells respond to all forces generated by heart contraction and blood circulation. They convert these electrical signals into biochemical signals and influence their own production of ECM as well as their interactions with other cell types. After an injury, cardiac fibroblasts are activated and relocate to the affected area, forming a scar. Dysfunction of cardiac fibroblasts can lead to the development of fibrosis, irregular heart rhythms and inflammation (Ghafouri-Fard et al., 2024).

Endothelial: Cardiac endothelial cells (CECs) have the ability to differentiate between activated cardiomyocytes and supporting fibroblasts. These elongated cells form a vital barrier within the arteries and ventricles and act as a crucial connection between the bloodstream and heart tissue. Together they help accelerate the heart rhythm. CECs make up the endothelium, the inner layer of blood vessels in the heart. They effectively regulate blood flow by facilitating both vasodilation and contraction, ensuring the efficient delivery of oxygen and nutrients to the heart while eliminating waste products. By expressing anticoagulant substances such as prostacyclin and nitric oxide, CECs play an important role in preventing the formation of blood clots. Maintaining a healthy heart requires striking the delicate balance between improving blood flow and inhibiting blood clotting. In addition, CECs have the ability to selectively allow immune cells and molecules to enter cardiac tissue, thereby exerting control over inflammation. CECs express specialized transporters that help transport glucose and fatty acids across cardiomyocytes, activate contractile mechanisms, and release various paracrine factors that communicate with neighboring cells such as cardiomyocytes and fibroblasts. These signals influence growth, survival and repair processes and maintain cardiac homeostasis. Although CECs do not produce direct electrical impulses, CECs contribute to cardiac function by influencing the formation of various channels and the expression of ion channels in neighboring cells. Aging, chronic diseases such as diabetes and hypertension, and exposure to toxins impair its function, causing arteriosclerosis, thrombosis, and blood deficiency (So et al., 2022; Chi and Song, 2023).

Smooth muscle: Unlike bone marrow and blood vessels, cardiac muscle cells (CSMC) do not suffer severe infection. Instead, they regulate the rhythm and maintain the balance of the cardiovascular system. CSMCs are found primarily in the walls of large arteries and play an important role in regulating blood flow in the heart. They control the diameter of the blood vessels through contraction and relaxation and ensure that the heart muscle cells are supplied with oxygen and nutrients. They also regulate lymphatic flow, promote proper elimination, and protect heart health. Although CSMCs do not produce direct electrical stimulation, they contribute to the propagation of electrical signals throughout the heart wall and influence the formation of various cardiomyocytes. They are particularly sensitive to changes in blood vessels and nerves. In response to injury or stress, CSMCs can differentiate into myofibroblasts and cause dense ECM deposition and fibrotic scarring. This condition disrupts vascular harmony and ultimately leads to heart failure. Inefficient CSMC causes chronic inflammation in the heart wall, recruits immune cells, and causes further effects of fibrosis and tissue damage (Hochman-Mendez et al., 2022). Aging and chronic diseases such as hypertension can impair CSMC function, cause vascular dysfunction, alter pressure regulation, and ultimately lower blood pressure. CSMC plays an important role in cardiac care. They regulate blood flow, prevent blood clotting, relieve pain and communicate with other cells in the heart. Many heart diseases can occur when the CSMC is damaged or destroyed (Benson et al., 2023).

Interstitial cells: Interstitial cells (ICs) are a dynamic and heterogeneous population of specialized cells with fibroblast-like morphology that present unique characteristics. Cardiac ICs are located within the ECM and CECs, which are associated with the great vessels, sinus endothelium, and cardiac endocardium, and fill the surface of the heart valve leaflets (Taylor et al., 2003). They synthesize non-cellular components of cardiac ECM including collagen, elastin, glycoproteins and proteoglycans, cytokines, growth factors, and chemokines. Therefore, they play a vital role in maintaining the three-dimensional structure of the ECM. Also, they induce the production of ECM remodeling enzymes, matrix metalloproteinases (MMPs), and tissue inhibitors (Chester and Taylor, 2007; Sanders et al., 2014). Additionally, cardiac ICs express many cardiac and smooth muscle (SMs)-specific markers such as the molecular marker α-actin (a marker of myofibroblasts), which helps to improve cardiac valve function. Thus, ICs can play a major role in the development of cardiac tissue engineering and maintaining cardiac ECM structure in pursuit of homeostasis (Forte et al., 2018).

## 3 Heart failure

The occurrence of cardiovascular diseases (CVDs) accounts for the majority of deaths worldwide, accounting for approximately 30% of all human deaths. Cardiovascular diseases include various diseases of the heart, blood vessels and brain. In 2017, 17.5 million people died from these diseases, with ischemic heart disease alone accounting for 4.7 million deaths. Unfortunately, statistical projections indicate a worrying trend: the expected mortality rate from cardiovascular disease is expected to increase significantly, from 17.5 million deaths in 2012 to a staggering 22.2 million in 2030 (Lacraru et al., 2023; Wasim et al., 2023).

The mortality rate from heart disease is significantly high due to the limited and inadequate ability of heart tissue to regenerate after damage. In ischemic heart disease, the blockage or narrowing of the coronary arteries impairs blood circulation and therefore the transport of vital oxygen and nutrients to the heart tissue. Insufficient oxygen supply ultimately leads to cardiomyocyte death, resulting in significant loss of functional myocardium in the affected area of the heart (Castillo-Casas et al., 2023).

**FIGURE 2:**
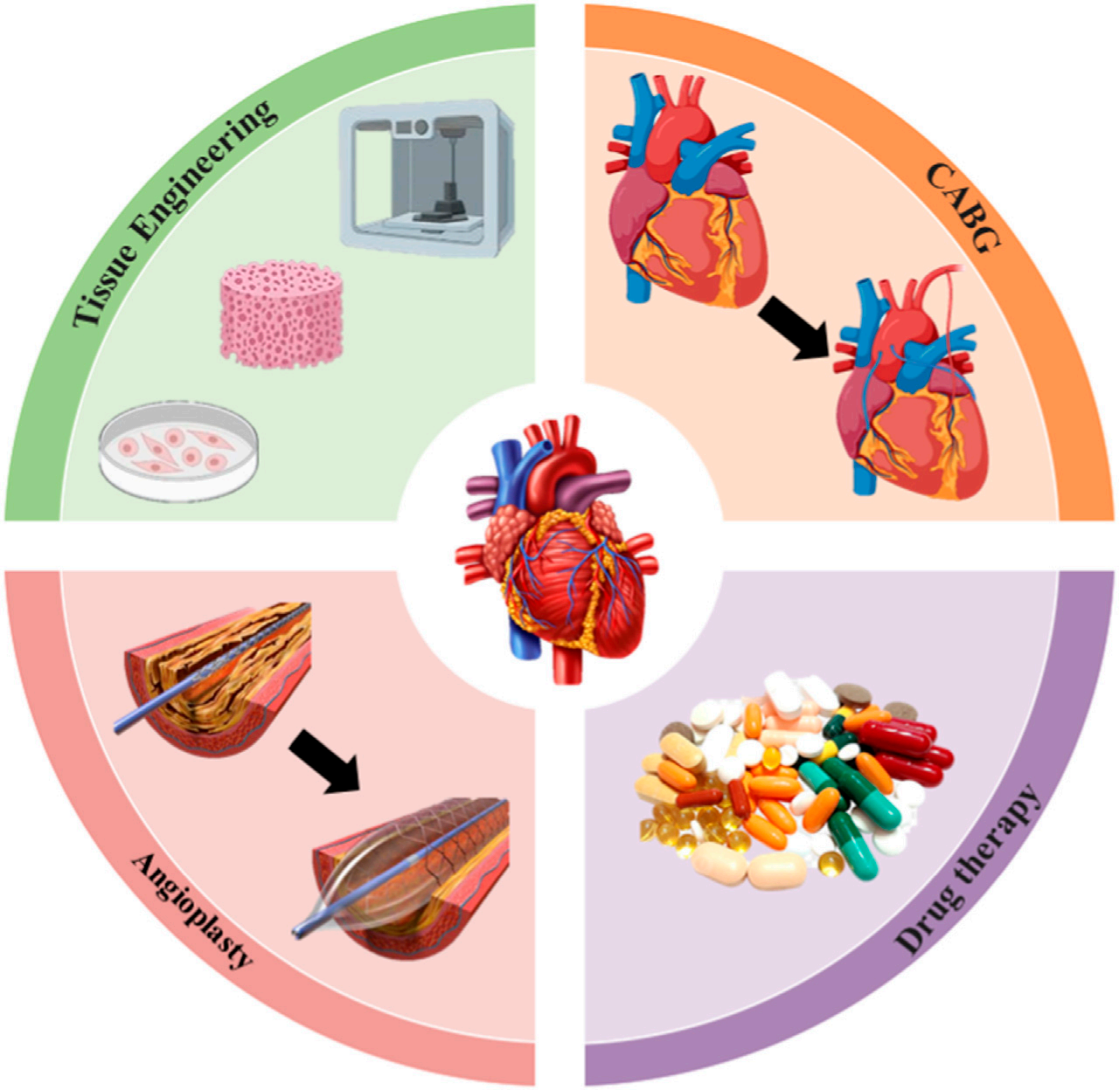
Strategies for the treatment of heart failure.

Advances in the field of medical, interventional and surgical cardiology have led to a significant decrease in mortality in the initial phase of myocardial infarction as well as an extension of the lifespan of patients. However, current remedies for heart failure have their limitations, whether in the early or advanced stages of symptoms, as they are unable to restore the affected heart (Seetharam et al., 2022). Existing treatments are mainly based on medication, surgery, left ventricular support devices and heart transplants. Medications that relieve stress on the heart (e.g., diuretics and blood thinners) and protect it from toxic humoral factors (e.g., β-blockers, spironolactone) are currently the standard conservative treatment (Spilias et al., 2023). While drug treatments can delay late-stage disease progression, they cannot prevent or reverse disease progression. Furthermore, more than 50% of patients with heart failure do not respond favorably to current drug treatments, demonstrating their limitations. Interventional treatments, including implantable pacemakers or surgery, improve patient survival but do not prevent disease progression to late stages or regeneration of dead myocardial cells (Maron et al., 2022). For people with severe heart failure, a heart transplant remains the only viable solution. Nevertheless, the shortage of donor organs and the side effects caused by the immune system response pose significant challenges in the treatment of patients with these diseases. Given this predicament, it is clear that it is imperative to promote the emergence of innovative therapeutic approaches that target them, to support the rejuvenation or regeneration of the heart (Table 1; Figure 2).

**TABLE 1:** Comparison of strategies for the treatment of heart failure.

Feature	Tissue engineering	CABG (coronary artery bypass grafting)	Drug therapy	Angioplasty
Approach	Regenerate damaged tissue with new cells and supporting structures	Bypass blocked arteries with healthy vessels from other parts of the body	Address symptoms and disease progression through chemical intervention	Open or minimally invasive procedure to widen narrowed arteries
Target	Advanced stage with significant tissue loss; specific tissue types	Severe blockages in major coronary arteries	Early to moderate stages; diverse range of CVDs	Moderate blockages, typically in single or few arteries
Efficacy	Long-term potential for complete tissue regeneration and restoration of function; still under development	Proven effectiveness in restoring blood flow and improving symptoms; carries surgical risks	Effective in managing symptoms and slowing disease progression, but cannot cure	Effective in widening blocked arteries and improving blood flow; potential for repeat procedures
Invasive	Highly invasive; requires surgery to implant biomaterials and cells. Or Minimally invasive; often outpatient procedure	Highly invasive; open chest surgery required	Moderately invasive; requires catheterization but no major incisions	Minimally invasive; often outpatient procedure
Risks and Complications	Infection, rejection of biomaterials, limited cell viability, tissue failure	Bleeding, infection, anesthesia risks, long recovery time	Side effects, adverse drug reactions, limited long-term benefits	Restenosis (re-narrowing), radiation exposure (with stents), potential for repeat procedures
Cost	High; still under development with limited availability	High; requires extensive surgery and hospitalization	Relatively low; medication costs vary	Moderate; procedure and stent costs vary
Long-term Outcome	Potential for complete cure and regeneration; research ongoing	Long-term symptom relief and improved quality of life; potential for re-blockages	Symptom management and slowing disease progression; may not prevent future complications	Temporary relief; often requires long-term medication or additional procedures

### 3.1 Drug therapy

Drug therapy can be helpful in relieving the symptoms of heart disease and improving the wellbeing of people with cardiovascular disease. It is imperative that patients adhere to the medication regimen set by their doctor and do not change the dosage without professional advice (Zaree et al., 2023). These medications include (Ali Meerza et al., 2021; Sakr et al., 2023):

• Blood pressure medications to lower blood pressure
• Beta-blockers to slow the heart rate
• Statins to lower cholesterol
• Pacemakers to help stabilize an irregular heartbeat
• Blood thinners to reduce or prevent blood clots in the coronary arteries
• Inotropic drugs to improve the heart’s pumping function
• Diuretics to remove excess fluids and reduce blood volume

Aspirin, also known as a remarkable drug, has the ability to reduce the risk of heart attacks or strokes in people who have already suffered a heart attack or stroke. In addition, it has been shown to be effective in maintaining open arteries in patients who have undergone coronary artery bypass graft surgery or other procedures to relieve arterial blockages, such as angioplasty. In emergency situations, aspirin is given to people who are suspected of having a heart attack or stroke. Still, aspirin’s potential to prevent heart attacks and strokes in people without a history of heart or brain disease has not yet been recognized by the Food and Drug Administration. In fact, consuming aspirin in particular can prove harmful without the risk of heart disease. Although aspirin has powerful properties, it is not without side effects. It can increase the risk of stomach ulcers, kidney and liver disease, and brain damage due to excessive bleeding. In addition, aspirin may have adverse interactions with other medications, leading to harmful consequences (Arnautu et al., 2022; Araujo et al., 2023; Wang F. M. et al., 2023). Clopidogrel, also called Plavix, is an antiplatelet drug used in people who suffer from heart attack or stroke, and blood circulation disease. Usually, it is used with aspirin to treat heart attack-induced worse chest pain, and keep blood vessels open to prevent blood clots after cardiac stent implantation and blood flowing confidently in the body. However, drug interactions may change the intended drug function or increase its side effects. Some drugs that can remove clopidogrel from the body include; tipranavir, omeprazole, fluvoxamine, fluoxetine, and others. Although the benefits of clopidogrel are greater than its drawbacks and any serious risk of side effects reported for it, patients may face several side effects such as stomach upset, low bruising or bleeding, constipation, and diarrhea. Despite these low side effects, clopidogrel is still used as an effective drug in the treatment of cardiovascular diseases (Lendaris et al., 2021; Kakamad et al., 2023; Chan et al., 2024).

Inotropic drugs are a class of drugs that affect the increase/decrease of the heart’s pumping power and are available in two types, positive and negative (Gustafsson et al., 2023). Inotropic drugs act on cardiomyocytes. Epinephrine (Adrenalin®), norepinephrine (Levarterenol®), dopamine, dobutamine, and levosimendan are positive inotropic drugs that increase heart rate, cardiac output, and blood/oxygen pumping to other organs by improving heart muscle contractions. These drugs are usually used in patients with congestive heart failure, pulmonary hypertension, bradycardia, and after open heart surgery. Despite the positive aspects of these drugs, side effects such as headache, high blood pressure, anxiety, fever, tachycardia or arrhythmia may occur after taking them (Juguet et al., 2020; Zheng Q. et al., 2023). Negative inotropic drugs, unlike their positive type, keep blood pressure at normal levels, reduce chest pain, regulate abnormal heart rhythm, and prevent hypertrophic cardiomyopathy by reducing heart muscle contraction. Disopyramide, atenolol, clonidine (or Catapres®), itraconazole (or Sporanox®), and verapamil (or Verelan®) can be mentioned among these drugs. As mentioned, negative inotropic drugs normalize blood pressure, help reduce chest pain, and improve cardiac rhythm problems. However, their use may be associated with the risk of side effects such as dizziness and headache, constipation, diarrhea, nausea, dry mouth, and blurred vision. Therefore, using inotropic drugs should be accompanied by a specific dose and a doctor’s prescription to help improve the individual’s life (Mehra et al., 2023; Willeford and Silva Enciso, 2023; Oricco et al., 2024).

### 3.2 Angioplasty

Angioplasty is an alternative treatment for cardiovascular disease. It is a minimally invasive procedure that opens narrowed or blocked arteries that supply blood to the heart muscle. First, the doctor numbs a small area of the groin or wrist. A narrow tube is then inserted into the artery to gain access to the coronary system. A remarkably thin wire is then inserted into the blocked area. This wire includes a catheter and a delicate balloon. The balloon is expanded, displacing the plaque and clearing the blockage. This facilitates smoother blood flow through the arteries. Once the catheter is removed from the patient’s hand or foot, the doctor or nurse applies pressure to the insertion site (Cappuzzo et al., 2022). Angioplasty does not require general anesthesia and is usually performed under local anesthesia or light sedation. Typically, angioplasty is completed within about an hour. In cases where multiple arteries are blocked, the procedure may take several hours. During angioplasty, the patient is given sedatives and intravenous blood thinners to prevent blood clots (Folberg et al., 2023). Angioplasty is a safe and effective treatment for coronary artery disease. It can relieve chest discomfort, improve heart function and reduce the risk of a heart attack. This treatment option is particularly suitable for people with coronary artery disease whose chest pain does not respond to medication. However, it carries some risks, including (Cao et al., 2022; Boudihi et al., 2023; Jin et al., 2023):

• Bleeding
• Infection
• Damage to the artery
• Recurrence of the blockage

### 3.3 Coronary artery bypass grafting (CABG)

Some patients may not be suitable candidates for angioplasty; Therefore, CABG surgery is recommended for them. If a person’s coronary arteries are narrowed or blocked so severely that the risk of a heart attack increases, a doctor may recommend CABG surgery. This type of surgery uses two different types of vascular grafts: venous and arterial. Venous grafts are harvested from the lower leg and thigh, while multiple arterial grafts can be used in CABG surgery (Tran-Nguyen et al., 2022; Wong et al., 2023). Typically, the left internal mammary artery (LITA), which is harvested from the inner wall of the left chest, is used in all patients. This particular artery has demonstrated the most favorable long-term results for CABG surgery and is typically used for the left anterior descending coronary artery (LAD), known as the main artery of the heart. The main concept of CABG surgery is to connect venous or arterial grafts to the narrowed or blocked arteries. This connection is achieved through suturing, scientifically known as anastomosis. The connection of the graft to the coronary artery is called a “distal anastomosis” because it represents the end point of blood flow in the graft. The blood supply to these transplants, which is ultimately directed to the coronary arteries, can be produced in three different ways. In the first method, the graft itself is connected to the existing blood supply network (so-called “*in situ* graft”), for example to the left internal mammary artery and, in certain cases, to the right internal mammary artery. The second method uses “free” venous or arterial grafts that must be connected to a blood supply source. In this case, the free grafts are connected through a hole at the level of the ascending aorta. Because this represents the starting point of blood flow in the graft, it is referred to as a “proximal anastomosis” (Kim J. S. et al., 2022; Saito et al., 2023; Takami et al., 2023).

### 3.4 Tissue engineering

**FIGURE 3:**
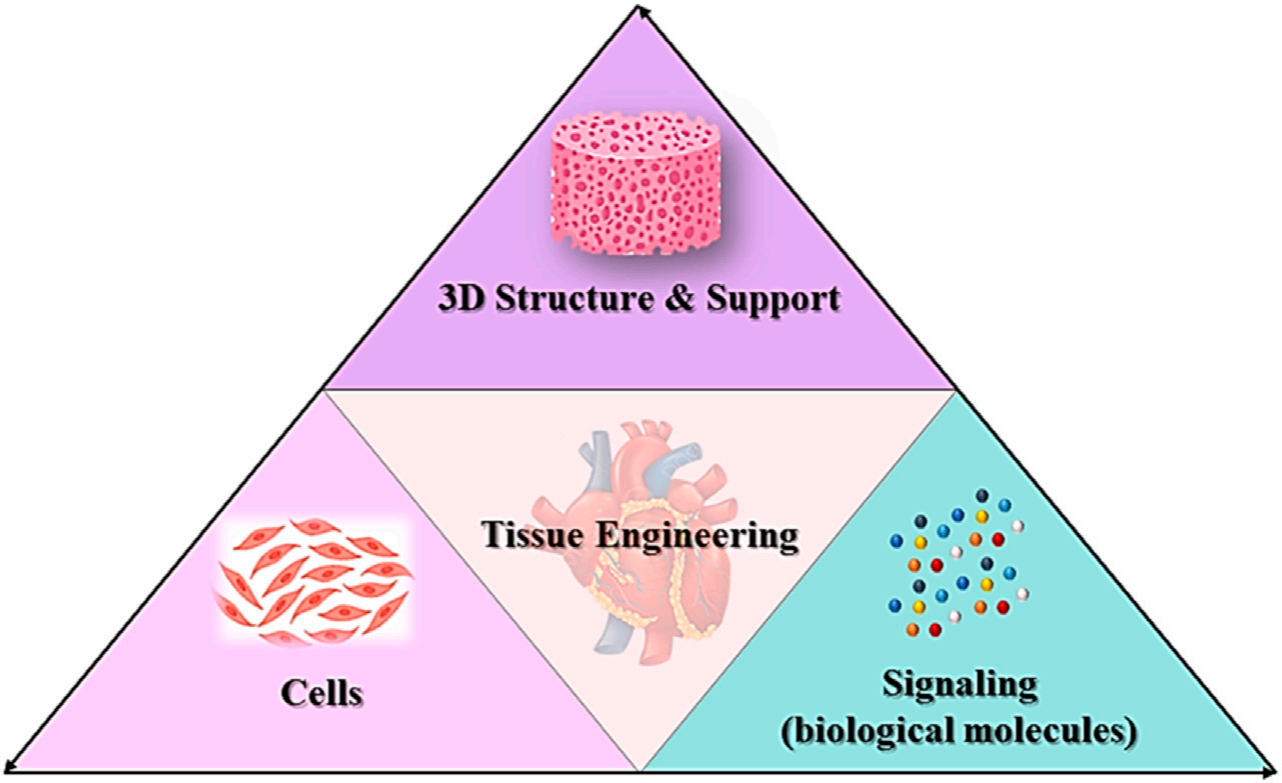
Principles of tissue engineering.

Tissue engineering is the implementation of a combination of cells, materials engineering methods and appropriate biochemical factors to improve or replace biological tissues (Figure 3). Tissue engineering uses a scaffold to construct a new and viable tissue for medical purposes (Nun and Joy, 2023; Soleymani and Naghib, 2023). Although most interpretations of tissue engineering encompass a wide range of applications, in reality it refers to applications that repair or replace a tissue (e.g., bone, cartilage, blood vessels, bladder, skin, muscle, etc.) (Zheng et al., 2021; Zhang Y. et al., 2022). Tissues often require mechanical and structural properties for optimal function. The term is also used to carry out specific biochemical purposes by deploying cells within an artificial support system (such as an artificial pancreas or an artificial liver) (Bonilla et al., 2021). The concept of tissue engineering is to create two different types of genetically modified tissue: one by growing cells in the laboratory on a scaffold and the other by implanting an acellular scaffold into the body that modifies the patient’s cells enabled. In both scenarios, the scaffold must be broken down along with tissue growth to ensure that as the tissue matures and grows, the scaffold no longer exists and the newly formed tissue can function similarly to the lost tissue (Regenberg et al., 2023). In tissue engineering, a porous structure is first prepared as an extracellular matrix or scaffold for cell growth and then growth factors are inserted. After sufficient growth of the cells in the voids, the scaffold is transferred from the laboratory environment to the living organism. Blood vessels gradually penetrate the framework and facilitate the nutrition of the cells. In the soft tissues of the organism, the framework is inevitably broken down and replaced by new tissue. However, materials that are not necessarily biodegradable can be used in rigid fabrics. Cultured cells can be tissue-specific or related stem cells. Stem cells are currently one of the most attractive research areas in biology. The reason for this can be determined by looking at the extraordinary properties of these cells. Basically, a stem cell is a cell with special properties that give it the ability to renew itself and differentiate into other cell types. This remarkable property of stem cells allows their use in regenerative medicine or cell therapy, justifying the extensive studies they receive in the field of tissue engineering (Snoeck, 2020; Ayavoo et al., 2021; Minh-Thai et al., 2021).

One of the most important concerns in tissue engineering applications is the patient’s immune system. The immune system plays an important role in regulating the systemic function and protection of the human body, which consists of a coordinated population of cells and imposes an inflammatory reaction on the implanted biomaterials (Tao and Wang, 2021). Therefore, the bulk materials and the materials created when a scaffold is dismantled must be biocompatible and degradable. It is of utmost importance that the selected shaping technique does not have a negative impact on the biocompatibility and biodegradability of the raw components of the framework. The primary goal of a scaffold is to direct and facilitate the movement and development of cells from adjacent tissues toward the site of injury or to promote the growth of cells transplanted onto the scaffold prior to transplantation. It is highly desirable that the surface possess chemical properties that promote cell adhesion and proliferation. Large pore diameter and high pore connectivity are essential for tissue formation and transport of nutrients and metabolic wastes. As porosity and pore diameter increase, this leads to an increase in the surface-to-volume ratio of the scaffold, i.e., pores (Chen Z. et al., 2022; Nazemi et al., 2023). For example, Chen and colleagues investigated the effect of porous structure and pore diameter of biodegradable poly (glycerol sebacate urethane) scaffold on cell proliferation, angiogenesis, and tissue growth. The pore size was reported between 6.4 and 28.1 μm and the scaffold porosity was 88%–96%. The results of cell culture on the scaffold showed an increase in metabolic activity and cell proliferation. In addition, the cells attached to the scaffold showed an increase in collagen deposition with an increase in porosity, which confirms its beneficial effect on cell behavior and tissue growth (Samourides et al., 2020). On the other hand, the role of softness or stiffness in increasing cell behaviors (growth, differentiation, proliferation) has been well investigated. It has been reported that a change in softness or stiffness of the scaffold leads to an increase in the tensile forces between cells and their entry into the cell cycle, and finally increases cell differentiation and proliferation. In addition, the damping property of the scaffold can affect cell behaviors by balancing the transmission of mechanical signals and increasing their mechanical properties such as resistance to shear forces (Yasodharababu and Nair, 2020; Omid et al., 2023). (Wei et al., 2020) reported that stiff hydrogel scaffolds can mimic osteoid stiffness and support bone stem cell differentiation. However, they may limit cell growth due to the dense matrix. Therefore, they developed soft and biodegradable hydrogel scaffolds mimicking the soft stiffness of bone marrow based on detachable matrix metalloproteinase (MMP), and RGD-based adhesive region to enhance cell proliferation. The results showed increased cell growth and proliferation in soft scaffolds. Furthermore, the culture of the scaffolds on the rigid bone-mimicking substrate showed that the cells migrate toward the interface and differentiate into bone cells (Wei et al., 2020).

In stem cell therapy, the most important limitations are transplantation, survival, and carcinogenesis in host tissue. Although the carcinogenic potential of the stem cell therapy approach in the heart field has not yet been determined, the ability of mesenchymal stem cells (MSCs) as carriers of cytotoxic agents to inhibit tumors has been proven. It should be noted that although MSC-based therapies for cancer are safe and beneficial, they have shown limited clinical efficacy (Vicinanza et al., 2022). For example, in a phase II clinical trial, the therapeutic effect of autologous MSCs and c-kit positive cardiac cells (CPCs) was evaluated in patients with heart failure. Patients were randomly subjected to transendocardial injection of MSCs/CPCs combination, CPCs alone, and MSCs alone for 12 months. Studies showed a significant improvement in patients’ quality of life after receiving the combination of MSCs + CPCs (*P* = 0.023), and MSCs alone (*P* = 0.050). Additionally, major adverse cardiac events (MACE) were significantly reduced in the presence of CPCs alone. It should be noted that no negative effects of stem cell injection or their carcinogenicity were reported in this study (Bolli et al., 2021). In the case of laboratory heart models for drug discovery and screening, toxicity testing, and disease modeling, the immaturity of cardiomyocytes derived from human pluripotent stem cells (hPSCs) as well as the lack of tissue-level information from standard two-dimensional (2D) culture environments remain major concerns issue (Maas et al., 2023). In this context, heart tissue engineering emerges as a promising strategy to address the above-mentioned problems and advance the field of heart disease treatment. Combining cardiac cells with biocompatible scaffolds can improve cell delivery accuracy and cell survival because the cells are accompanied by a mechanical support element. Furthermore, by mimicking the cardiac environment through the use of advanced signaling systems, cells are exposed to important regulatory signals not present in standard 2D culture environments. Therefore, the immature hPSC-CM phenotype can develop into a quasi-mature phenotype (Dhahri et al., 2022). In this regard, Lou et al. developed cardiac patches containing four cell types differentiated from hPSC-CM to investigate cardiac recovery after myocardial infarction. The results of histological and echocardiographic analyses of MI mice hearts after 4 weeks reported reduced infarct size, better grafting, and significant improvement in cardiac function (Lou et al., 2023).

In the field of heart tissue engineering, the original goal was to create analogues of cardiac tissue to repair or replace damaged myocardium. Today, the main goal of the scientific community is to overcome the main obstacles to this approach before clinical implementation. However, diverse laboratory applications for cardiac tissue, such as safety pharmacological assessments and disease modeling, have become increasingly important. This means transferring laboratory research to the clinic and treating patients. The basic design approaches for tissue engineering may include only cellular constructs, cells in combination with biomaterials, or only biomaterials. However, three elements are often essential for producing reliable three-dimensional cardiac structures that resemble cardiac tissue (Wu et al., 2020; Alizadeh et al., 2021; Han et al., 2023; Rana et al., 2023):

i. A large number of cardiac cells to achieve physiological density.
ii. Scaffolds made from biocompatible materials that mimic the three-dimensional environment.
iii. Biomimetic signaling systems, which may include signaling molecules and other mechanical and electrical regulatory signals.

#### 3.4.1 Cardiac cells

The first challenge in tissue engineering is to find suitable cell sources in appropriate quantities. To date, the majority of work in the field of producing three-dimensional heart tissue analogs has been carried out using rat or laboratory mice and newborn mouse hearts. However, due to recent advances in the field of human stem cells, the field of cardiac tissue engineering is gradually shifting away from engineering cardiac tissue using animals and instead focusing on applying the knowledge gained to develop human cardiac constructs (Salem et al., 2022; Hong et al., 2023). Human pluripotent stem cells, including both hESCs (human embryonic stem cells) and hiPSCs (human induced pluripotent stem cells), are considered the most promising cell source for restoring human cardiac function and creating human cardiac models. When obtaining hESCs, cells are taken from the inner cell mass of a fertilized human embryo in the early stages of development. These cells have the remarkable ability to divide indefinitely, allowing them to be cultured and propagated in large quantities. Through the action of specific extracellular signals, hESCs can differentiate into different cell types. Similarly, hiPSCs exhibit numerous properties similar to hESCs, such as their ability to proliferate indefinitely and the potential to differentiate into multiple lineages. However, they are obtained from differentiated adult cells through the forced expression of specific genes. Therefore, hiPS cells eliminate the ethical and legal problems associated with hES cells and can produce cardiac cells that are genetically equivalent to a given patient’s cells. For these reasons, hiPSCs are a very good source of cardiomyocytes for cardiac tissue engineering approaches (Nit et al., 2021; Isaja et al., 2022; Tian et al., 2023).

Most existing differentiation protocols for hESCs use two-dimensional culture systems. Although two-dimensional differentiation produces the desired cell lineage and allows direct characterization of cells during the differentiation process, this type of cell culture does not take into account the importance of the entire three-dimensional cell environment (Savoj et al., 2022). Two-dimensional systems can enhance unnatural interactions with associated extracellular factors (e.g., multiple cell types, extracellular matrix, cytokines, and physical factors) that can alter or influence cellular responses. The idea of designing biomimetic models in two- and three-dimensional form is to exploit the full potential of stem cells by reproducing some aspects of *in vivo* physiology, since the signals transmitted to cells are the main determinants of their phenotype (Monteiro et al., 2023).

#### 3.4.2 Biomaterials

The extracellular matrix (ECM) surrounding cardiac cells provides environmental signals and mechanical support to cells that control cell growth and function. Therefore, to produce reliable three-dimensional cardiac tissue constructs, it is essential to use biological materials that exhibit the important features of cardiac ECM, such as an anisotropic fiber structure, mechanical properties and a body-like molecular composition tissue (Derrick and Noel, 2021; Hu et al., 2022). Biological substances serve as supporting structures that facilitate attachment, organization, and maturation of cardiac cells or act as vehicles for cell delivery. These organic components and structures can be used in either solid or gel form and can be manufactured in various shapes and sizes. If we focus on scaffolds used in the creation of cardiac tissue constructs, these can be made from natural substances such as collagen, Matrigel, and chitosan or synthetic materials such as poly (glycerol sebacate), poly (glycolic acid), and polycaprolactone. In addition, a combination of natural and synthetic types can be used (Apsite et al., 2020; Raghav et al., 2022; Tevlek et al., 2022).

##### 3.4.2.1 Natural biomaterials

Natural biological materials have the appropriate molecular composition for cell adhesion, survival, and differentiation. They are biodegradable and can be broken down *in vitro* and *in vivo* within a few days or weeks. They are converted into non-toxic degradation products by cellular enzymes, and cells can break down these materials and replace them with their own ECM components (Yazdanian et al., 2022). Despite all these advantages, it should be noted that natural biological materials have weak mechanical properties and have limited access to structural modifications and elastic moduli in the range of 10 Pa to 100 kPa (Liu H. C. et al., 2023). They also have different physicochemical properties because they come from different protein sources and may contain surface antigens that can trigger an immune response. However, this is not a major obstacle as there are sources of ECM-derived biological materials approved for human use (Patrawalla et al., 2023). Various components of the ECM (proteins, glycosaminoglycans, glycoproteins, and small molecules) can be isolated and prepared for use in scaffold fabrication. The most common are collagen (most abundant protein in the body), alginate (a polysaccharide obtained from brown algae), chitosan (a polysaccharide obtained from crab, lobster, and shrimp), Matrigel (a combination of ECM proteins and small molecules), fibrin (a fibrous protein formed from fibrinogen) and gelatin (a collagen extracted protein) (Table 2) (Sonmezer et al., 2023). ECM can also be used as a scaffold after being decellularized from the heart or its various parts. The main advantage of this method is that it provides the appropriate environment for cell growth by taking into account the *in vivo* macroscopic and microscopic structure of the heart as well as its extracellular components. The decellularization process does not damage the vascular channels and the newly cultured cardiac cells show contractile activity in the matrix (Ghiringhelli et al., 2021; Wang D. et al., 2023). However, for heart tissue sections, the orientation of the section is important because different cutting planes result in different scaffold structures and pore sizes. Furthermore, it should be taken into account that the decellularized matrix from adult hearts may lack some growth factors important for controlling stem cell differentiation or cardiomyocyte maturation (Belviso et al., 2022).

**TABLE 2:** Common natural biological materials for use in scaffold fabrication.

Material	Source	Properties	Features	Ref
Collagen	Skin, bone, cartilage, tendon	Biocompatible, biodegradable, good mechanical strength	Fibrous structure, has various types, including collagen types I, II, III, IV	Zhao et al. (2021)
Alginate	Brown algae	Biocompatible, biodegradable, electrically conductive	Gel-like structure, has a negative charge, can be polymerized with divalent ions such as calcium	Zhang et al. (2021a); Mangalath et al. (2023)
Chitosan	Animal exoskeletons, brown algae	Biocompatible, biodegradable, anti-inflammatory, anti-cancer	Linear polymeric structure, has hydroxyl and amino groups, can be polymerized with divalent ions such as calcium	Rahman et al. (2020); George and Shrivastav (2023); Yu et al. (2023)
Matrigel	Hexapod tissue	Biocompatible, biodegradable, contains a wide range of signaling molecules	Complex structure, contains various types of proteins, carbohydrates, and small molecules	Alsaab et al. (2022); Batool et al. (2022); Gianni and Jemth (2023)
Fibrin	Blood	Biocompatible, biodegradable, good mechanical strength	Fibrous structure, contains fibrin and fibrinolysis proteins	Johari et al. (2022); Kumar et al. (2022); Risman et al. (2023); Zhao et al. (2023)
Gelatin	Skin, bone, cartilage, tendon	Biocompatible, biodegradable, good mechanical strength	Gel-like structure, contains various types of collagen proteins	Kim et al. (2021); Wang et al. (2024)

##### 3.4.2.2 Synthetic biomaterials

Synthetic biomaterials are manufactured (unlike natural biomaterials) through fully controlled processes, which gives them the ability to customize their mechanical properties, topography, structure, biocompatibility and biodegradability. Consequently, they can be produced in a predictable and reproducible manner (Duceac and Coseri, 2022). However, synthetic biomaterials often lack the ability to support cell adhesion and survival, requiring the addition of appropriate bioactive molecules to achieve the desired functionality (Ballard et al., 2024). Furthermore, the implantation of these biomaterials into the body may encounter certain obstacles, including the induction of inflammatory responses, erosion, insufficient biocompatibility, inability to integrate into host tissue, or the production of degradation products that are not completely excreted from the body (Table 3) (Frazao et al., 2020). Various synthetic polymers have been used for the fabrication of three-dimensional scaffolds in cardiac tissue engineering applications. The most commonly used include polyurethane (PU), polycaprolactone (PCL), polylactic acid (PLA), polyglycolic acid (PGA), poly (glycerol sebacate) (PGS) and their copolymers (Wu et al., 2021; Fukunishi et al., 2022; Rodriguez-Merchan, 2022). For example, when primary cardiac cell cultures from newborn rats are seeded onto electrospun polyurethane scaffolds with aligned fibers, they exhibit characteristics of a mature phenotype (Chen Y. et al., 2022). Similarly, by combining poly (glycerol sebacate) scaffolds with newborn rat heart cells and appropriate electrical and molecular signals, contractile cardiac structures with maturation-related features can be obtained (Wu et al., 2023). In other studies, polycaprolactone nanofibrous scaffolds have been shown to increase adhesion and electrical coupling between different layers of the heart and vessels of newborn rats, and PLLA electrospinning scaffolds support the growth and proliferation of rat myocytes better than polylactic acid scaffolds with other polymers (Lv et al., 2022).

**TABLE 3:** Comparison of characteristics and properties between Natural Biomaterials and Synthetic Biomaterials (Fornasari et al., 2020; Khan et al., 2022; Choi et al., 2023).

Feature	Natural biomaterials	Synthetic biomaterials
Source	Derived from biological sources (animals, plants, microorganisms)	Produced through chemical synthesis or fabrication
Biocompatibility	Generally biocompatible; low risk of immune response	Biocompatibility varies; some require modification for compatibility
Biodegradability	Biodegradable, mimicking natural tissue turnover	Biodegradable and non-biodegradable options available
Mechanical Properties	Can have weak mechanical properties, depending on source	Often have stronger mechanical properties, but can be less flexible
Cell Adhesion and Growth	Often provide good cell adhesion and support for growth	Require modification to promote cell adhesion and growth
Vascularization	Can promote natural vascularization due to existing ECM components	May require additional strategies for vascularization
Cost	Can be expensive, depending on source and purification	Often cheaper to produce than natural materials
Controllability	Properties can be variable, difficult to control precisely	Properties can be more precisely controlled and tuned
Examples	Collagen, Matrigel, Chitosan, Fibrin	Polyurethane, Polycaprolactone, Polylactic Acid, Polyglycolic Acid

#### 3.4.3 Signaling

The growth and development of the heart are controlled by the interaction of numerous biochemical and biophysical signals that take place in a three-dimensional environment. It takes several years for human cardiomyocytes to reach their fully mature form in terms of their dimensions, structure, molecular composition, energy utilization, and general physiological capabilities within the organism (Kim Y. et al., 2022; Singh et al., 2023). To generate functional human heart structures *in vitro*, biochemical, mechanical, and electrical stimuli are integrated into cell culture systems via bioreactor or microphysiological system settings (Castro et al., 2020). Tissue structures are engineered to have characteristic features of the heart muscle, such as B. anisotropic cell orientation (for proper signal propagation), coupling, synchronized contractions at physiological rates, responsiveness to cardiac electrical stimuli, and efficient exchange of nutrients and metabolites between cells and their environment. Various physiological factors influence the development of hPSC-CMs, both in terms of their structure and function (Song et al., 2023; Takahashi et al., 2023). These crucial factors include a variety of elements, such as:

• Interaction with other cell types: hPSC-CMs interact with other cell types such as endothelial cells and fibroblasts to form functional cardiac constructs. The exchange between the units facilitates the advancement of the development of hPSC-CMs and the construction of a complex network of blood vessels (Vargas-Valderrama et al., 2022; Wan et al., 2022).
• Substrate stiffness: The substrate stiffness on which hPSC-CMs are cultured may influence their maturation. The presence of softer substrates promotes the proliferation of hPSC-CMs, while stiffer substrates facilitate their differentiation (Kumarapuram et al., 2022). The accuracy of this issue has been proven in previous studies (Wei et al., 2020). (Dattola et al., 2019) prepared 3D PVA-based scaffolds and investigated their potential for the growth and proliferation of hPSC-CMs. The stress-strain curve of the scaffold showed the same elastic behavior as the muscle ECM. SEM images of pore diameter in the range of 10–370 μm (corresponding to the dimensions of the cells) showed that it creates a suitable substrate for cell proliferation. Observations of cell proliferation after 12 days of hPSC-CMs culture showed an increase in the number of cells compared to the initial numbers, as well as an increase in the expression of anti-troponin T (cardiac sarcomere specific marker), which confirmed the cell compatibility of the substrate (Dattola et al., 2019).
• Long-term culture: Long-term culture of hPSC-CMs can also promote their maturation. This could potentially be due to the accumulation of epigenetic changes that stimulate differentiation (Tsoi et al., 2022). For example, Floy and colleagues differentiated hPSCs into epicardial cells (EpiCs) and cardiac progenitor cells (CPCs) to investigate the effect of their coculture (2 weeks) on cardiomyocyte proliferation and cardiac repair. The results showed that in the presence of TGFβ inhibitor A83-01, EpiCs remain in an epicardial state and induce cardiomyocyte proliferation. In addition, increased expression of cTnI and MLC2v was observed in developing cardiomyocytes, which led to a decrease in sarcomeres organization (Floy et al., 2022).
• Three-dimensional environment: The most common method for producing cardiac tissue structures is to culture them into shapes that define the three-dimensional structure of the scaffold. Hydrogels containing cardiac cells are often used for this strategy. There are various methods for building three-dimensional environments, such as using permeable scaffolds, empty heart tissue, layered cell layers, modular multilayer assembly, and 3D printing. When hPSC-CMs are cultured in a three-dimensional environment, they have a higher chance of reaching maturity compared to those cultured in a two-dimensional environment. This phenomenon is thought to occur due to the increased surface area to volume ratio and the existence of extracellular matrix (ECM) molecules that facilitate differentiation (Gu et al., 2021; Shojaie et al., 2021; Zhang M. et al., 2022). For example, in one study, the therapeutic effects of several biochemical factors such as dexamethasone, thyroid hormone, and insulin-like growth factor-1 (IGF-1) on the maturation of hPSC-CMs after culture in 3D cardiac microenvironments were investigated. The results proved that the presence of IGF-1 supplement in the 3D microtissue creates a higher fidelity adult cardiac phenotype (Huang et al., 2020).
• Biochemical factors: Biochemical factors such as growth factors and cytokines can also influence the maturation of hPSC-CMs. Growth factors such as fibroblast growth factor 2 (FGF-2) stimulate the expansion and specialization of hPSC-CMs. Cytokines such as transforming growth factor β (TGF-β) have the ability to facilitate the conversion of hPSC-CMs into specific cell categories such as fibroblasts or endothelial cells (Jin et al., 2020; Alex et al., 2023).
• Cell patterning and alignment: Cell patterning and alignment may also influence the maturation of hPSC-CMs. hPSC-CMs cultured in a structured environment are more likely to align in the same direction, which is important for the formation of functional cardiac tissue (Dark et al., 2023).
• Mechanical stimulation: Mechanical stimulation, such as stretch or compression, can also promote the maturation of hPSC-CMs. By subjecting hPSC-CMs to mechanical stimulation, their growth and differentiation can be enhanced, thereby leading to an improvement in their contractile function. This process aids in the promotion of optimal cellular development and functionality (Nguyen-Truong et al., 2020; Johnson et al., 2024). In this regard, (Leonard et al., 2018) designed a system for the culture of engineered heart tissues (EHT) that adjusts the mechanical afterload conditions. After 3 weeks of culture, it was found that the applied afterload increases the length of sarcomeres, increases the area and length of cardiomyocytes, improves calcium handling, and increases the expression of several key markers of cardiac maturation, such as fetal ventricular myosin heavy chain isoforms (Leonard et al., 2018).
• Electrical stimulation: Electrical stimulation can coordinate and activate the intrinsic forces associated with cell contraction, and then regulate tissue structure and function of CMs. Furthermore, it causes the opening of calcium channels through which many intracellular signaling pathways are regulated. Therefore, electrical stimulation can be useful as an endogenous method to improve the growth and maturation of hPSC-CMs (Zhang R. et al., 2021). To investigate the effect of sustained electrical stimulation on the properties of EHTs produced from neonatal rat heart cells (rEHT) and hPSC-CMs (hEHT), Eschenhagen et al. developed a new pacing system. The researchers found that over 16–18 days of continuous application of 0.5 Hz frequency, rEHT produced a greater force (×2.2) than unstimulated rEHT. They also observed increased expression of connexin-43, improved sarcomere structure, rising rate of Ca^2+^ curve, and increased cardiac cell density in the center of EHT. In addition, for hEHT after 14 days with a frequency of 1.5 to 2 Hz, a force increase of 1.5 times was observed compared to unstimulated hEHT. In addition, increased cytoplasm-to-nucleus ratio, and improved muscle network of cardiomyocytes proved that sustained electrical stimulation could enhance the maturation properties of hPSC-CMs (Hirt et al., 2014).

The integration of these diverse parameters is essential for the development of functional hPSC-CMs.

## 4 Application of tissue engineering in heart failure

Tissue engineering researchers are using biological 3D printing techniques to create structures that repair, reinforce, or replace vessels damaged in heart failure. For example, vascular replacement or strengthening is used to treat a variety of conditions, including the correction of aortic aneurysms and inherent anomalies. Building on the discourse on cardiac tissue and its associated diseases and hurdles, research was conducted into the creation and configuration of three-dimensional scaffolds in the field of cardiac tissue engineering (Rioux et al., 2022; Zheng Z. et al., 2023).

Cardiac cells require a complex environment to grow and function normally. Providing an optimal substrate for cardiac cell growth, survival, and electrical interactions without toxicity is a key challenge in cardiac tissue engineering (Gomes et al., 2022). Electrically conductive cardiac tissue engineering scaffolds can be used to treat heart disease. For example, electrically conductive scaffolds can be used to repair damaged heart tissue or redirect electrical impulses in the heart. Various types of electrically conductive materials have been investigated for use in cardiac tissue engineering. These materials include conductive polymers, metallic nanomaterials and carbon nanomaterials. The conductivity of three-dimensional structures is crucial for electrical signaling, adhesion and networking of cardiomyocytes, cell maturation and cell differentiation in cardiac tissue engineering (Lee et al., 2022; Roacho-Perez et al., 2022).

**FIGURE 4:**
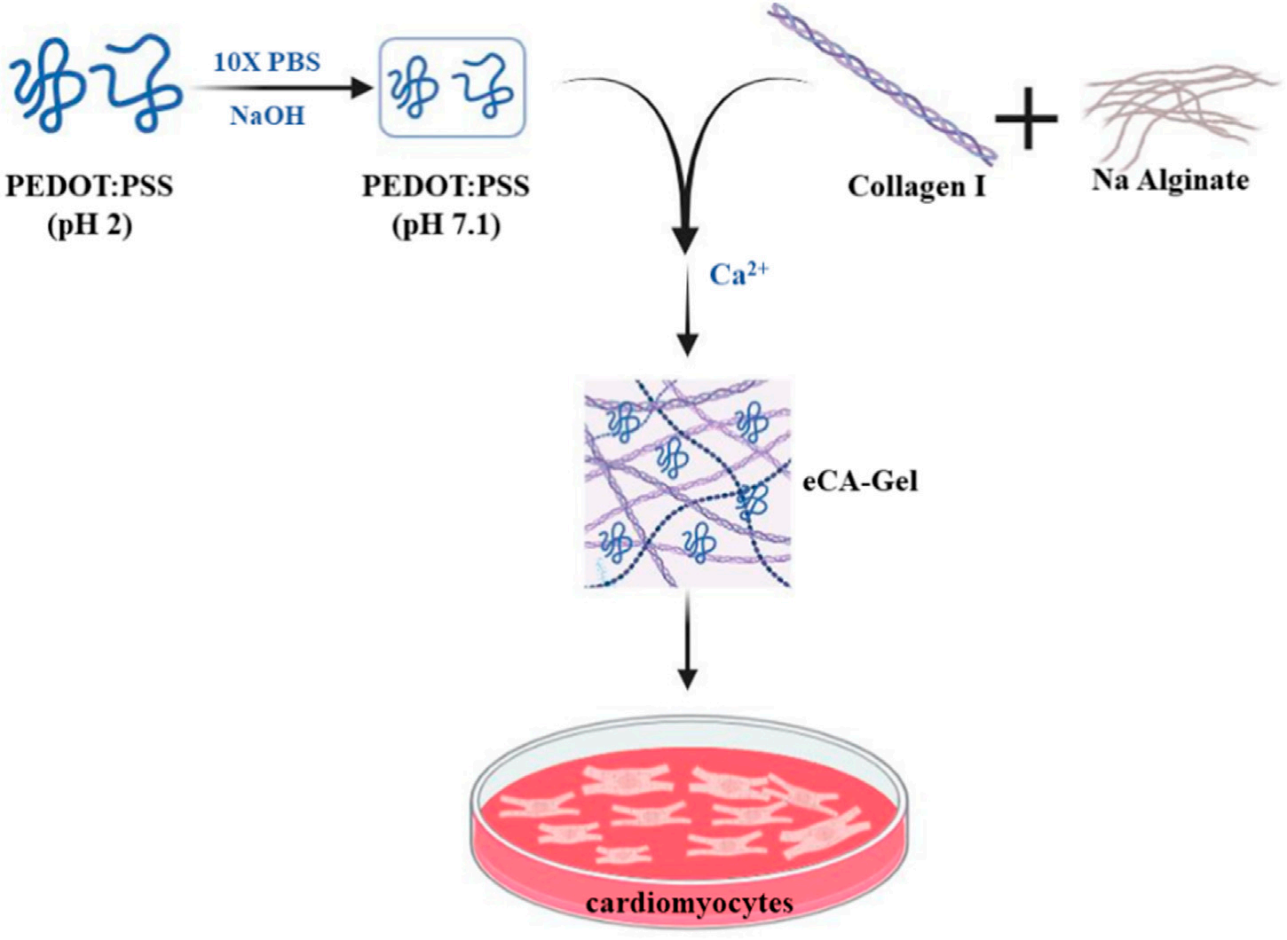
The steps of making eCA-Gl hydrogel for evaluation on cardiomyocyte cells.

Roshanbinfar et al., 2018 developed a hydrogel scaffold made of collagen, alginate, and poly (4,3-ethylenedioxythiophene); Polystyrene sulfonate (PEDOT:PSS) was studied using solution casting techniques to investigate its ability to mimic the fibrous structure of the extracellular matrix (ECM), improve electrical connectivity, and promote cardiomyocyte maturation. This scaffold was prepared by dissolving PEDOT:PSS in PBS solution and then adding collagen and sodium alginate and finally adding calcium chloride as a cross-linking agent of the final hydrogel. Two samples with different proportions of PEDOT:PSS (0.26 and 0.52 wt%) were examined. When studying the swelling of hydrogels, it was observed that the swelling percentage increased with increasing PEDOT:PSS amount from about 4,000% to about 2,000% and this value corresponds to the combination of different percentages of conductive polymer in both samples. Shear stress in the sample at 0.52 wt% The value of the conductive polymer was highest at about 40 Pa, and the elastic modulus for this sample was also calculated to be about 230 Pa. After culturing cardiomyocytes on hydrogels and performing characterization on day 13 of culture, it was observed that cell viability and proliferation were the same in both conductive hydrogels, but a higher frequency in the 0.52 wt% sample was intended for the induction of cell beating. On the other hand, the expression of Connexin-43 and Troponin T genes was higher in the sample with 0.52 wt% conductive polymer (Figure 4) (Roshanbinfar et al., 2018). The results of this study show that the use of PEDOT:PSS in collagen-alginate hydrogels creates an electrically conductive system with a structure similar to natural interstitial tissue. This new hydrogel is cytosolic and increases the physiological rate of cardiomyocytes in neonatal rats while reducing internal arrhythmias. In addition, manipulated cardiac tissue can be controlled by external electrical stimulation. This tissue consists of self-contracting hearts with dense structures of elongated cardiomyocytes. It was also observed that iPSC-derived cardiomyocytes in these electrically conducting hydrogels had sarcomere length, contraction speed, contraction amplitude, and synchronous beating similar to those of mature human cardiomyocytes. These results suggest that these hydrogels accelerate the maturation of iPSC-derived cardiomyocytes. As a result, a new approach is presented to produce engineered heart tissue that can beat and mature without external stimulation and that can be used in drug screening or in the production of tissue therapies.

Delivery of exogenous nitric oxide (NO) to the infarcted myocardium has been shown to be an effective strategy for the treatment of myocardial infarction due to the diverse physiological functions of NO, but reperfusion of blood flow to ischemic tissues is accompanied by overproduction of toxic reactive oxygen species (ROS), which can worsen tissue damage and reduce the effectiveness and efficiency of the therapeutic approach (Hao et al., 2022a; Qian et al., 2023; Li et al., 2024). In one study, an injectable hydrogel of boronic acid-protected diazeniumdiolate-modified chitosan (CS-B-NO) with shear-thinning properties was synthesized for MI treatment (Hao et al., 2022b). This hydrogel releases NO in response to ROS stimulation and has dual ROS scavenging and NO releasing functions. It also modulates ROS/NO imbalance after ischemia/reperfusion (I/R) injury. The CS-B-NO hydrogel was injected *in situ* into the ischemic myocardium of a mouse model and showed the desired therapeutic efficacy in contrast to hydrogels with only NO or ROS scavenging properties. In the early stage of I/R injury, it reduced myocyte apoptosis and inflammatory response. Long-term treatment with CS-B-NO hydrogel promotes cardiac repair and improves cardiac function. Furthermore, the hydrogel protects the heart from oxidative stress by enhancing Keap1-S-nitrosylation to activate the Nrf2 signaling pathway and reducing IKK/IκBα-mediated phosphorylation of the NF-kB signaling pathway. The absence of Nrf2 reduces the antioxidant capacity of the hydrogel both *in vitro* and *in vivo*, indicating the cardioprotective effect of the hydrogel.

**FIGURE 5:**
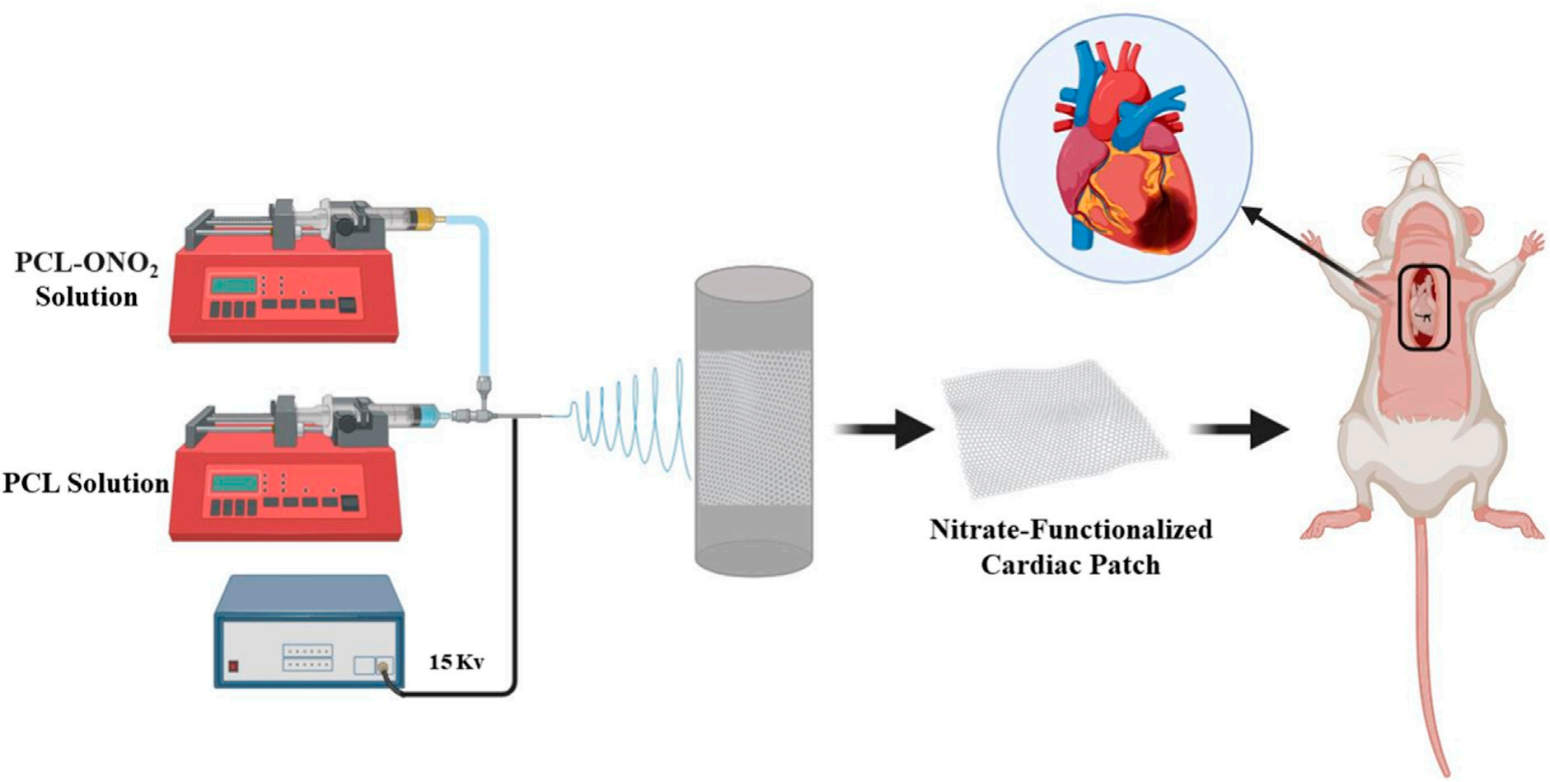
Electrospun cardiac patch with gradual NO release.

In another study, a nitrate-functionalized cardiac patch was produced that releases nitric oxide (NO) directly into cardiac tissue instead of traditional nitrate medications (Zhu et al., 2021a). These medications often cause side effects. In contrast to these drugs, in this patch the pharmacological nitrate groups are directly linked to biodegradable polymers, effectively converting a small molecule drug into a therapeutic biomaterial (Zhu et al., 2021b). NO, as a versatile signaling molecule, plays a crucial role in regulating cardiovascular homeostasis and can prevent infarction formation by relaxing vascular tone, inhibiting platelet aggregation, and modulating the inflammatory response, thereby exerting a cardioprotective effect (Tian et al., 2022). Dysregulation of the NO signaling pathway is always associated with increased myocardial infarction. In this study, short-chain PCL oligomers with nitrate-capped ends (PCL-ONO2) were first synthesized by reaction with bromobutanoyl chloride and substitution of the terminal bromine with silver nitrate. Then, PCL-ONO2 was mixed with high molecular weight PCL in the ratio of 1:9, and electrospun nanofibers were obtained by applying 15 kV (Figure 5). The results showed that the mechanical properties exhibited a moderate decrease in tensile strength and strain rate due to the presence of PCL-ONO2 and on the other hand also improved the surface hydrophilicity by reducing the contact angle. When implanted onto the myocardium, the patch locally and gradually releases NO. Due to the ischemic (oxygen-poor) environment in the injured area of the heart, NO production is significantly increased in this area. This process protects heart cells from damage and improves heart tissue repair. The results of this study demonstrate the high potential of this functional patch for the treatment of ischemic heart disease through a mechanism different from traditional nitrate drugs.

**FIGURE 6:**
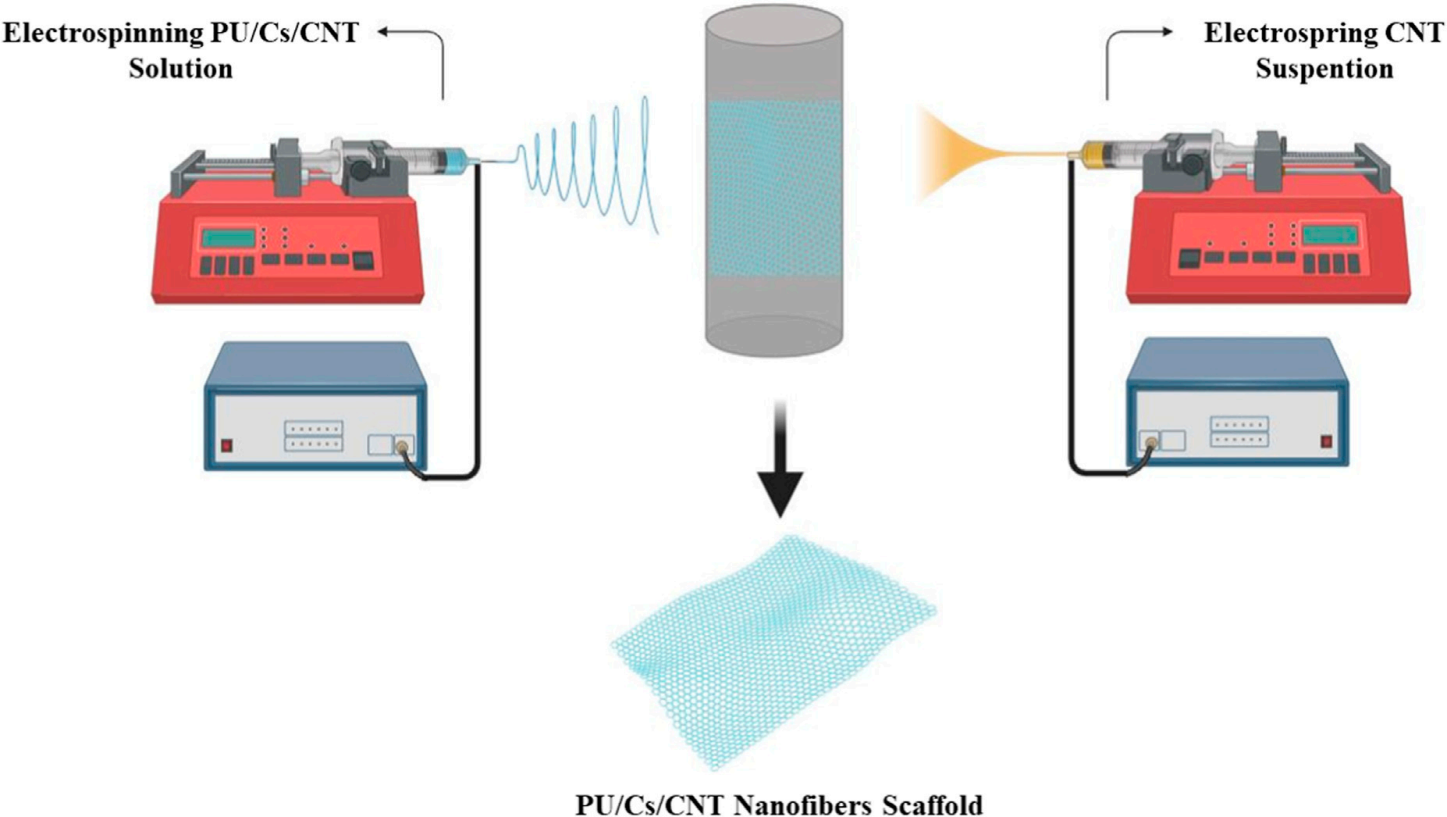
Cardiac PU/Cs/CNT nanofiber scaffold by simultaneous electrospinning and electrospraying method.

In one study, an electrostatic and biodegradable scaffold was fabricated. Polyurethane (PU)/chitosan (Cs)/carbon nanotubes (CNT) composite nanofibers with random and aligned orientation were electrospun to structurally mimic the extracellular matrix (ECM) with a flow rate of 1 mL/h and an applied voltage difference of 20 kV. The scaffolds were fabricated by simultaneously electrospinning PU/Cs and electrospraying a 1% (w/v) CNT dispersion in ethanol. The results of evaluation of the fabricated scaffold showed that these scaffolds are both biocompatible with H9C2 cells and electron conductive. Carbon nanotubes (CNTs) are two-dimensional materials with excellent mechanical, electrical and thermal properties. In this study, it was demonstrated that CNTs, as an electrostatically conductive filler, imparted electrostatic properties to the scaffolds. Furthermore, the prepared structures showed proper wettability and mechanical properties for cardiac tissue engineering. Furthermore, the conductive electrostatic nanofiber scaffolds demonstrated suitable biocompatibility to support cell attachment and proliferation (Ahmadi et al., 2021). The results obtained demonstrate the potential of this scaffold for cardiac patch applications (Figure 6).

One method that has received considerable attention in tissue engineering for vascularized organs is the use of scaffolds in which autologous cells can be cultured. The researchers’ main goal is to create an environment with a three-dimensional extracellular matrix and the patient’s specific cell types to create a functional tissue that reduces the risk of immunogenicity. The use of this method for vascular structures has been explored using a variety of scaffold biomaterials such as synthetic polymers, natural polymers, and decellularized animal and human tissues. Some studies failed due to a lack of cell infiltration or a lack or weak acceptance of the artificial organ. In addition to cell culture on a scaffold, other manufacturing techniques for creating tubular and vascularized organ structures have also been described since the 1980s. Weinberg and Bell pioneered tissue engineering of blood vessels in 1986 using smooth muscle cells in a collagen-containing scaffold and vascular endothelial fibroblasts. However, their system required the integration of an artificial Dacron network to increase mechanical strength. This limitation was attributed to the composition of the extracellular matrix and the low cell density (Weinberg and Bell, 1986). An advance in this field was then made by (L’heureux et al., 1998) who used a method that did not involve the use of artificial or exogenous biological materials and produced the desired tissue by inducing collagen secretion in smooth muscle and fibroblast cell culture plates. The layers of these tissues were then wrapped around a rod and later cultured as a tube to resemble the final artery containing the separate layers of endothelial, medial and external vascular layers (L’heureux et al., 1998).

**FIGURE 7:**
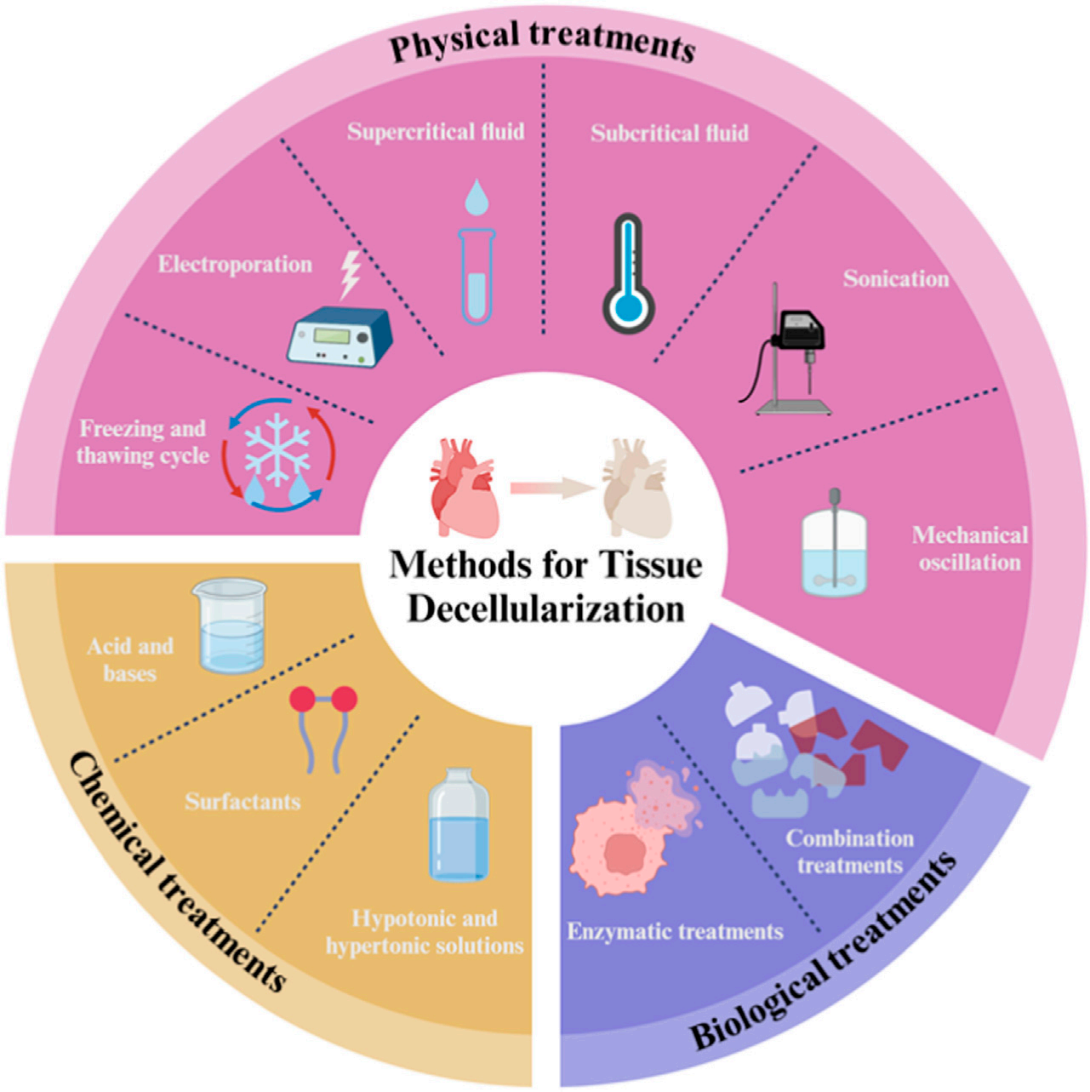
Tissue decellularization methods.

Decellularization offers a more efficient option for reducing the burden of autologous transplantation than other vascular tissue engineering (VTE) technologies. This approach produces much more accurate copies of ECM with superior bioactivity, immunogenicity and biodegradability and relies on a variety of physical, chemical and biological approaches or combinations of these individual approaches to disrupt cell membranes through immersion, perfusion and agitation conditions (Figure 7). Decellularization strategies vary greatly depending on the different characteristics of the tissue, including structure, components, size and thickness. Theoretically, most cellular epitopes and antigens that trigger immune responses and contribute to graft failure are eliminated after vascular decellularization. As a result, decellularization leads to the production of optimized non-immunogenic vascular analogues whose ECM is comparable to the native structure. Such ECM scaffolds can provide a suitable environment for cell integration/differentiation amenable to *in vitro* vasculogenesis (Yamanaka et al., 2020; Shojaie et al., 2021; Marvin et al., 2022; Dehghani et al., 2024).

Adult mammals are unable to regenerate functional cardiac tissue, but newborn mammals are capable of robust cardiomyocyte proliferation and regeneration within a week of birth. Given this shift in regenerative capacity through growth, the extracellular matrix (ECM) from adult tissues may not be beneficial for promoting cardiac regeneration. A study evaluated the potential of neonatal mouse cardiac ECM (nmECM) to prevent maladaptive ventricular remodeling in adults using an *in vivo* acute myocardial infarction (MI) model. The hearts of newborn mice were isolated and then decellularized with an antibiotic-antifungal and gentamycin solution (A/A/G). MI was induced in mice by occlusion of the left anterior descending coronary artery. Immediately after injury, nmECM was injected directly into the injured area. The results showed that significant improvements in cardiac function were observed after a single administration of nmECM, while amECM did not improve these parameters. Treatment with nmECM limited scar extension in the left ventricle and induced neovascularization in the injured area. nmECM has also been shown to induce the expression of the ErbB2 receptor, which simulates a neonatal environment. Thereby, it promotes cardiac function associated with neuregulin-1, and inhibition of the ErbB2 receptor effectively prevents these effects, demonstrating its role in the context of nmECM as a treatment. This study demonstrated the *in vivo* potential of a neonatal-derived biomaterial to prevent widespread ventricular remodeling in adult mice following myocardial infarction (Wang et al., 2019). This study makes a clear and convincing case for the potential of nmECM to treat heart failure, but is limited to mice and it is not yet known whether the results translate to humans. Additionally, nmECM has not been compared to other possible treatments for heart failure. The results suggest that nmECM has the potential to be a promising new treatment for this disease.

In another study, the myocardial gene expression pattern was evaluated after myocardial infarction (MI) in a standardized rodent LAD ligation model with and without ventricular stabilization using a tailored dECM-based cardiac scaffold (cdECM) (Aubin et al., 2022). MI was induced in male Wistar rats by standard LAD ligation. Cardiac ECM from donor rats was then used to fabricate individual tissue-engineered myocardial sleeve (TEMS) cdECM scaffolds. Cardiac function was assessed after 4 and 8 weeks. Ventricular stabilization led to integration of the TEMS scaffold into the myocardial scar with varying degrees of cellular infiltration and also significantly improved echocardiographic parameters, suggesting attenuation of maladaptive cardiac remodeling. Furthermore, TEMS implantation after MI altered the myocardial gene expression pattern, with differences in gene expression evident at 4 weeks with a significant decrease in the expression of NPPA, NPPB and PDGFB and an increase in the expression of IL-10 and IGF1. However, after 8 weeks, differences in gene expression patterns of markers associated with remodeling and angiogenesis were still observed between groups. As shown in this study, ventricular stabilization by TEMS implantation prevents progressive left ventricular dilation after myocardial infarction, and rats with supported ventricles by TEMS implantation after LAD ligation not only had smaller LVEDDs compared to their control group at 8 weeks, but also comparable LVEDDs values at the time of TEMS implantation 14 days after myocardial infarction. Furthermore, the TEMS scaffold is not only extensively infiltrated by host cells but also appears to integrate into the scar area after myocardial infarction. Therefore, a biological interaction with the host tissue likely plays a role that goes beyond the mechanical one.

Adverse cardiac remodeling is characterized by biological changes that affect the composition and architecture of the extracellular matrix (ECM), leading to impaired signaling that can affect the balance between the cardiac and profibrotic phenotype of cardiac progenitor cells (CPCs). Belviso et al. The aim was to compare the effects of human-derived decellularized ECM (dECM) from normal (dECM-NH) or failing (dECM-PH) hearts on CPCs by culturing CPCs on dECM segments. When cultured on dECM-NH, CPCs significantly increased cardiac commitment markers (CX43, NKX2.5), cardioprotective cytokines (bFGF, HGF), and the angiogenic mediator NO. When cultured on dECM-PH, CPCs upregulated proregenerative cytokines (IGF-2, PDGF-AA, TGF-β) and the oxidative stress molecule H2O2, and culture on dECM-PH was associated with impaired paracrine support of angiogenesis and increased Expression of the KDR/VEGFR2 isoform of the vascular endothelial growth factor (VEGF)-sequestering receptor. The results showed that CPCs exposed to the pathologically remodeling ECM microenvironment partially lose their paracrine angiogenic properties and release more profibrotic cytokines. These results demonstrate the interplay between ECM and stromal CPCs, which justifies the cautious use of non-healthy decellularized myocardium for cardiac tissue engineering approaches. The study suggests that ECM from failing hearts may promote a profibrotic phenotype in CPCs. This is likely due to the presence of increased levels of profibrotic cytokines and other factors in the dECM-PH. These factors may signal CPCs to become more myofibroblastic, which can lead to scarring and impaired cardiac function. The findings of the study are important for the development of new therapies against heart failure. By understanding how the ECM can impact CPCs, researchers may be able to develop strategies to prevent or reverse the profibrotic phenotype in CPCs (Belviso et al., 2020).

**FIGURE 8:**
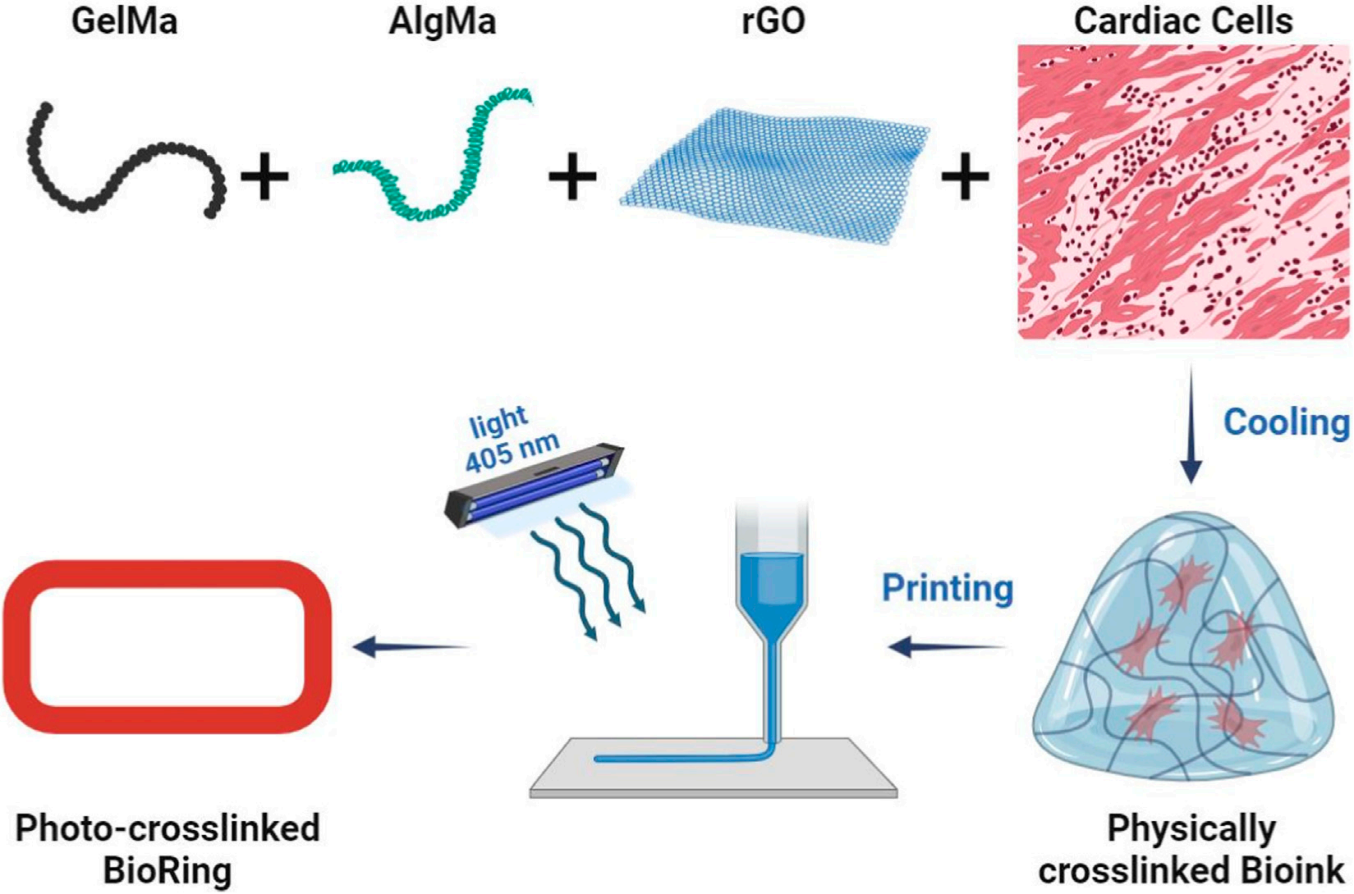
Preparation process of GelMA-AlgMA-rGO bioink and BioRing print for cardiac modeling.

Another modern method for creating 3D structures in tissue engineering is the use of 3D printers in biomedical applications. Recent advances in microfabrication techniques have made 3D bioprinting a layer-by-layer approach to create structures with higher precision and repeatability and control over geometric complexity, control over density and cell viability with cell penetration and high adhesion efficiency and porosity (Guimaraes et al., 2022; Rothbauer et al., 2022; Ming et al., 2023). The bioink used should be formulated based on biocompatible, bioactive and biodegradable materials with appropriate physicochemical properties to mimic the tissue microenvironment and regulate cell growth and function (Yazdanian et al., 2022). In one study, novel composite bioinks based on GelMA, AlgMA and rGO were developed and optimized for the cardiac platform on a 3D bioprinting chip (Figure 8) (Mousavi et al., 2024). Composite bioink components based on natural cross-linkable polymers, methacryloyl gelatin (GelMA) and alginate methacrylate (AlgMA), and reduced graphene oxide (rGO) electrically conductive nanomaterials were synthesized and characterized, and bioinks were formulated with different concentrations. The photocross-linked bioinks exhibit an interconnected porous microarchitecture with detectable rGO nanosheets on the pore walls. They added GelMA, AlgMA and rGO bioinks to improve the mechanical tensile and compressive properties, rheological properties and printability of the inks. The addition of these two substances also controlled the destruction characteristics of the structures. Subsequently, these bioinks were 3D printed in a ring-shaped structure (hereinafter “BioRing”) with different types of cardiac cells (newborn rat hearts, cardiac fibroblasts and HL-1 cells) and then subjected to biological evaluation. The results of the optimized bioink evaluation demonstrated high levels of cardiac cell viability and proliferation, as well as targeting functional markers (such as transient and spontaneous calcium). This printed model can be used for various applications including drug screening and heart disease modeling due to its similar and similar properties to real heart tissue.

In recent years, tissue engineering has become an important and attractive approach in the study of heart disease. The diversity of biomaterials and the way they are used have given rise to different strategies. Table 4 summarizes some of the studies conducted in recent years.

**TABLE 4:** Some research on the application of tissue engineering in cardiac diseases.

Fabrication methods	Materials	Animal models	Therapeutic effect	Refs
Hydrogel	PLA-PEG-NIPAAM	Rats	stimulated vascularization and Reduced infarct size	Zhao et al. (2020)
	Matrigel ™- HA-Alginate	Rats	Improved Cardiac functions	Tan et al. (2020)
	Chitosan-DEX-β-glycerophosphate	Rats	Increased EF and vessel density and decreased the fibrotic area and infarct size	Hua et al. (2020)
	Chitosan	Mice	Improved cardiac function	Liu et al. (2020)
	Collagen-carbon nano tubes-chitosan-gold nanoparticles	—	Non-toxic, promising heart tissue engineering biomaterial	Nezhad-Mokhtari et al. (2020)
	Collagen-chitosan composite scaffold	—	Biocompatibility, strong expression of cardiac-specific proteins, compact material, high porosity (>65%), and good mechanical properties within the physiological range of native myocardium are the hallmarks of CM.	Liu et al. (2023b)
	Chitosan-vitamin C based	—	Improve cardiac function	Guo et al. (2022)
Electrospinning	polypyrrole -chitosan-polyethylene oxide	—	Cell adhesion and proliferation and growth, Conductive	Zarei et al. (2021)
	Polyurethane-CS-carbon nanotubes composite	—	Biocompatibility, electrical conductivity, and potential for future use	Zadeh et al. (2021)
CELLULAR PATCHES	Fibrin-Thrombin- hiPSC-CM-EC-MSCs	Swine	Reducing infarct size and improving cardiac function	Gao et al. (2020)
	Fibrin - hiPSC-CMs	Rabbit	Improving heart function and reducing scar size	Jabbour et al. (2021)
	PECUUS - porcine dECM	Rats	Neovascularization in the peri-infarct area and improve cardiac function	Kashiyama et al. (2022)
	Polyaniline- Gelatin - PVA	Rats	Cardiac remodeling and prevention of ventricular fibrosis	Yu et al. (2022)
	Porcine dECM	Rats	Promoting neovascularization and improve cardiac function	Kim et al. (2022c)
	Fibrin - Engineered microvessels	Rats and swine	Promoting neovascularization and Reducing scar	Su et al. (2020)
	GelMa - PGDA	Mice	Increasing the rate of scologenesis and cell transplantation	Cui et al. (2020)
ACELLULAR PATCHES	GO - Silk fibroin	Rats	Repaired the infarcted myocardium	Zhao et al. (2022)
	PCL	Rats and swine	Reduced cardiac injury, suppressed adverse cardiac remodeling and ameliorated heart function	Zhu et al. (2021a)
	GO - PVA	Mice	Reduced myocardial fibrosis and promoted neovascularization	Yang et al. (2023)
	GelMA	Rats	Improved cardiac function and reduced fibrosis	Chen et al. (2022c)
3D printing	Non-mulberry silk fibroin Human umbilical vein endothelial cells (HUVECs)–in the bioink	—	Electrical conductivity	Mehrotra et al. (2021)

## 5 Future outlook

According to the studies reviewed, cardiac tissue engineering has been shown to provide a revolutionary approach to the treatment of heart failure, but significant barriers to widespread clinical application remain. The key limitations in cardiac tissue engineering include cell limitation and maturation, scaffold design and vascularization, electrical integration and *in vivo* maturation, and finally personalized medicine and affordability. Research into the use of induced pluripotent stem cells (iPSCs) promises personalized and ethical cell sources. Developing bioreactor systems to promote *in vitro* maturation and mimic the cardiac environment could improve performance (Chang et al., 2021; Roshanbinfar et al., 2021; Tadevosyan et al., 2021; Laschke and Menger, 2022; Hong et al., 2023). Current methods for obtaining functional cardiac cells are limited. There are ethical concerns with embryonic stem cells, while adult stem cells often have lower regenerative potential. Artificial tissue often has difficulty achieving the full maturation and function of natural heart muscle. (Redpath and Smart, 2021; Tenreiro et al., 2021; Kuchakzadeh et al., 2024). On the other hand, developing biocompatible scaffolds that mimic the complex structure and mechanical properties of natural heart tissue is still challenging. Integrating functional vasculature into engineered tissue is critical to ensure proper blood flow and nutrient delivery, but has not yet been fully implemented. It is very important to design biocompatible and biomimetic scaffolds that closely resemble the natural tissue of the heart. Advanced 3D bioprinting techniques with bioinks containing cells, biomaterials and growth factors can create complex and well-defined heart structures (Badria et al., 2020; Zakko et al., 2020; Szklanny et al., 2021; Jones et al., 2023). Seamless electrical integration of engineered tissues with the recipient heart requires further development to achieve optimal synchronous contraction and pumping performance. Current *in vitro* maturation methods may not fully recapitulate the physiological environment of the heart, potentially limiting function after implantation. Electrical integration strategies and improved *in vivo* maturation techniques are currently being investigated. These could include bioreactors that mimic the electrical and mechanical environment of the heart in preparation for preimplantation. The use of patients’ own tissue promises personalized therapeutic strategies and disease models to understand the specific causes of heart failure. Combining genetically engineered tissues with gene therapy, cell therapy, or biotechnological approaches may provide synergistic benefits to improve cardiac repair and regeneration. Minimally invasive artificial tissue insertion techniques facilitate implantation and improve patient outcomes (Dessauge et al., 2021; Pretorius et al., 2021; Yin et al., 2023; Cofino-Fabres et al., 2024). Finally, developing engineered tissues for individual patients using their own cells is a future goal, but technical challenges and potential cost limitations need to be addressed. By addressing these limitations and pursuing future directions, cardiac tissue engineering has the potential to revolutionize the treatment of heart failure and give patients hope for improved quality of life and a potentially viable heart replacement.

## 6 Conclusion

The frequency of heart failure increases with age. Approximately 10% of people age 65 and older suffer from heart failure. Most people treated for heart failure are able to live long and fulfilling lives. The field of tissue engineering holds enormous potential for transforming the approach to treating heart failure. By developing fully functional heart tissue, there is the possibility of repairing or replacing damaged tissue, improving heart function and reducing the need for heart transplants. Ongoing research is currently exploring multiple tissue engineering options in the context of heart failure. The techniques used in tissue engineering, such as the use of materials and the creation of three-dimensional structures, are very diverse. The current study included a comprehensive evaluation of various methods for structure creation, including the use of stem cells, biomaterials and 3D printing. Stem cells are undifferentiated cells with the ability to develop into different cell types and can be obtained from various sources such as the heart, bone marrow and adipose tissue. By differentiating stem cells into cardiomyocytes, the building blocks of the heart muscle, it is possible to manipulate heart tissue. Biomaterials, on the other hand, serve as supporting materials that promote the growth and maturation of cells. They can serve as scaffolds for cell growth and also serve as carriers for the delivery of drugs or other cardiac-targeted therapeutics. The emergence of 3D bioprinting technology has opened new horizons, allowing the creation of three-dimensional structures based on precise digital models. This technology can be used to produce scaffolds for tissue engineering purposes as well as custom design of cardiac implants. While tissue engineering to treat heart failure is still in its early stages, its potential to revolutionize disease treatment cannot be underestimated. Given the ongoing commitment to research and development, it is plausible that tissue engineering could 1 day provide a definitive cure for heart failure.

## Abbreviations

ICD, implantable cardiac defibrillators; MEF, mechanical feedback mechanism; ECM, extracellular matrix; CECs, Cardiac endothelial cells; CSMC, cardiac muscle cells; ICs, Interstitial cells; CVDs, cardiovascular diseases; LITA, left internal thoracic artery; hPSCs, human pluripotent stem cells; hESCs, human embryonic stem cells; hiPSCs, human induced pluripotent stem cells; NO, nitric oxide; ROS, reactive oxygen species; CNT, carbon nanotubes; VTE, vascular tissue engineering; nmECM, neonatal mouse heart-derived ECM; CPCs, cardiac progenitor cells.
